# Diet-Driven Variations in Longevity and Fecundity of the Endangered Tiger Beetle *Cicindela anchoralis* (Coleoptera: Carabidae)

**DOI:** 10.3390/insects16101066

**Published:** 2025-10-18

**Authors:** Deokjea Cha, Anya Lim, Jong-Kook Jung

**Affiliations:** 1 Insect & Invertebrate Restoration Team, Division of Restoration Research, Research Center for Endangered Species, National Institute of Ecology, Yeongyang-gun 36531, Republic of Korea; cj34gun@nie.re.kr; 2 Restoration Research & Assessment Team, Division of Restoration Research, Research Center for Endangered Species, National Institute of Ecology, Yeongyang-gun 36531, Republic of Korea; ppardus08@gmail.com; 3 Department of Forest Environment Protection, Kangwon National University, Chuncheon 24341, Republic of Korea

**Keywords:** *Cicindela anchoralis*, protein-to-carbohydrate ratio, prey-insect preference, insect conservation

## Abstract

Diet is a key factor modulating the trade-off between fecundity and longevity, a cornerstone of life-history theory. While laboratory studies have demonstrated that high-protein-to-carbohydrate (P:C) ratio diets increase reproductive output at the cost of lifespan, it remains unclear how this trade-off operates in species exposed to natural dietary variability and prey choice. We tested whether diet-mediated trade-offs between fecundity and longevity are modulated by prey-insect type in the endangered tiger beetle, *Cicindela anchoralis*, a species with a short adult lifespan. Tiger beetles were offered a choice between a high-P:C diet (cricket) and low-P:C diet (ant). Tiger beetles consuming the high-P:C diet exhibited increased fecundity and reduced longevity, while those feeding on the low-P:C diet showed the opposite pattern. Despite these consequences, both sexes showed a consistent preference for the high-P:C diet, suggesting that beetles prioritize reproductive output over lifespan. These results suggest that prey-insect selection might be an adaptive way to boost reproductive success within a limited adult lifespan, which may raise tiger beetles’ intrinsic rate of natural increase. Our findings highlight the ecological relevance of diet-driven life-history trade-offs and offer practical guidance for mass propagation strategies to support endangered tiger beetle recovery.

## Simple Summary

We studied how prey type affects reproduction and lifespan in the endangered tiger beetle *Cicindela anchoralis*. Tiger beetles fed on crickets (high-P:C) produced more eggs but lived shorter, while those fed on ants (low-P:C) lived longer but reproduced less. Despite this trade-off, beetles of both sexes preferred crickets. This indicates a life-history strategy prioritizing reproduction over longevity. Our findings highlight the ecological significance of diet-driven trade-offs and provide practical guidance for conservation programs. By tailoring diets, breeding efforts can enhance reproductive success, supporting population recovery of this critically endangered insect.

## 1. Introduction

The quality of the diet consumed by organisms has a significant effect on their fecundity and longevity [1,2,3]. In particular, the ratio of protein to carbohydrates (P:C) in the diet plays a critical role in shaping these life-history traits in *Drosophila* [4]. Notably, low-P:C diets are associated with extended lifespan, whereas high-P:C diets tend to shorten lifespan across various organisms [5,6,7,8], suggesting that responses to nutritional changes may represent a fundamental life-history adaptive strategy. Regarding the impact of these nutritional changes on lifespan and fertility, there are two conflicting explanations existing for the effects of high- and low-P:C diets. First, an example of adaptive resource re-allocation, which is commonly applied to low-P:C diets, posits that under dietary restriction (i.e., limited nutrient intake without malnutrition), organisms reallocate resources from reproduction to somatic maintenance, thereby enhancing survival until environmental conditions improve [9,10,11,12]. Second, as an example of detoxification, certain amino acids, such as those found in high-P:C diets, are known to increase fertility but shorten lifespan due to their toxic effects [6,13]. Understanding these feeding-related life-history strategies may be important for conserving endangered species through ex situ conservation methods. Specifically, conservation success depends not only on restoring population numbers but also on aligning restored habitats with the nutritional and ecological conditions that optimize both longevity and reproductive output [14,15].

Although laboratory studies on various insects, including red flour beetle (*Tribolium castaneum*) [16], rice leafroller (*Cnaphalocrocis medinalis*) [17], lesser Eucalyptus longhorn beetle (*Phoracantha recurva*) [18], and oriental fruit fly (*Bactrocera dorsalis*) [19], have supported diet-mediated life-history adaptive strategies, relatively few studies have examined these mechanisms under natural dietary conditions [20]. This gap needs attention because management interventions based on incomplete or artificial understandings of nutritional trade-offs could inadvertently reduce survival or fecundity in reintroduced populations. Moreover, there is growing concern that some life-history patterns observed in laboratory-reared insects, *Drosophila*, under non-stressful conditions may be artifacts that do not occur under actual wild conditions [21,22].

In this study, we examine *Cicindela anchoralis* (Chevrolat, 1845) (Coleoptera: Carabidae: Cicindelinae) [23], a critically endangered predatory insect in South Korea, as categorized in the national Red Data Book [24]. We aim to test whether the observed longevity–fecundity trade-offs in this species, which appear to be linked to dietary choice, represent life-history adaptive strategies shaped by ecological pressures or whether they are merely artifacts of controlled laboratory conditions. Specifically, we evaluate variations in the longevity and fecundity of *C. anchoralis* using two prey insects with different P:C ratios under circadian temperature and light similar to their habitat. To explore this, we tested four predictions: (*prediction I*) the amount of prey insects consumed would vary depending on the sex of the tiger beetle; (*prediction II*) tiger beetles increase intake of low-P:C diet (ant) over high-P:C diet (cricket) to compensate for nutritional deficiencies; (*prediction III*) longevity and fecundity of tiger beetles are modulated by two prey insects under circadian temperature and light conditions; and (*prediction IV*) tiger beetles prefer a certain type of diet (ant or cricket) rather than the balanced diet (ant with cricket) by selecting among prey to achieve maximum fecundity. Finally, we discuss the implications of these findings for developing captive breeding strategies in conservation programs for endangered tiger beetles.

## 2. Materials and Methods

### 2.1. Experimental Insect-Rearing Conditions

We used adult *C. anchoralis*, a critically endangered predatory insect that inhabits beach dunes, for the experiments. This species mainly hunts juvenile crabs, beach flies, and various invertebrates, or scavenges on dead organisms during the summer period [25,26]. Adult tiger beetles are active for approximately 40–50 days from mid-June to early August in South Korea. To assess longevity and fecundity, beetles were collected at the beginning of their activity period in mid-June 2024 from Shinan (34.792996° N, 125.968496° E), South Korea.

All collected adult tiger beetles were then individually reared during the experiment in plastic jars (120 mm in diameter and 80 mm in height) filled with sea sand. Soil moisture was maintained at 12% by misting approximately 1 mL of water onto the soil surface once daily, providing surface moisture for adult hydration and a moist substrate for oviposition. Laboratory conditions were adjusted to mimic the natural summer circadian patterns in tiger beetles’ habitats, using 100 W heat bulbs to maintain daytime temperatures to 28 °C and gradually lowering it to 23 °C at night under a 16:8 light–dark cycle. Relative humidity was maintained at 50–60% by spraying water once daily.

Adult tiger beetles used in the experiment were captured from their natural habitat, and only virgin individuals were selected to measure fecundity (the total number of eggs produced by the female) and lifespan (the lifespan of both sexes). Virginity was defined as follows: (i) females that had not laid any eggs during the first two-week rearing period, and (ii) males that did not display any mating behavior when kept with a female during the first two weeks of the rearing period. Females that laid eggs and males that exhibited mating behavior in the first two-week rearing period were excluded from the experimental subjects. Excluding non-virgin individuals, ten tiger beetles consisting of five pairs of male and female insects were used for each experiment. Because *C. anchoralis* is designated a Class I endangered species by the Ministry of Environment of South Korea, collection and research was conducted under permit from the Yeongsan River Basin Environmental Office (Permission No. EK202405ECP0001).

### 2.2. Prey Insects

To investigate the relationship between prey insects and the fecundity–longevity trade-off in tiger beetles, we selected two insect species with contrasting nutritional profiles (high and low P:C ratios), both of which occur naturally in the tiger beetles’ diet. The P:C ratio in diet is expressed as the ratio of the carbohydrate content relative to the protein content of the diet [27]. In this experiment, the nutritional composition of 100 g of the prey insects (ant and cricket) was analyzed externally by the Korea Standard Test and Analysis Research Institute (Ansan-si, South Korea), and the P:C ratio was calculated by dividing the total protein content by the total carbohydrate content. The workers of the Japanese wood ant (*Formica japonica*) species were used as the low-P:C diet (P:C; 3.28:1). The 2nd instar of the two-spotted cricket (*Gryllus bimaculatus*) was selected as the high-P:C diet (P:C; 6.06:1) (Table 1). Both prey insects were selected not only for their contrasting nutritional profiles but also for their comparable body size, thereby minimizing potential biases in prey acceptance or handling efficiency due to size differences.

### 2.3. Experiments

**Figure 1:**
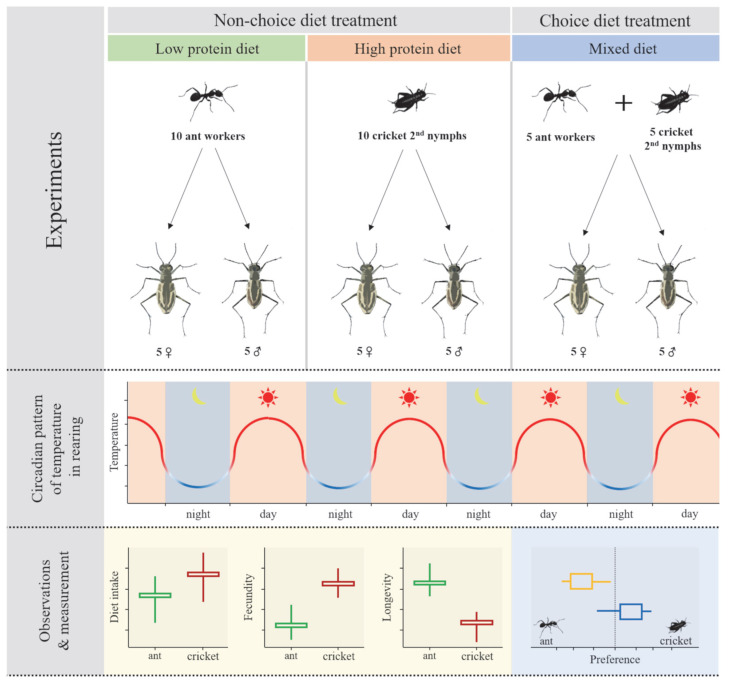
The diagram of the experimental procedure for observing variations in life-history traits according to prey insects under conditions with a circadian pattern of temperature and light.

To examine the life-history adaptive strategies of tiger beetles under different dietary conditions, we conducted two independent feeding experiments: a non-choice diet treatment and a choice diet treatment. Both experiments were carried out simultaneously under the prey-insect and rearing conditions described above (Figure 1).

#### 2.3.1. Experiment 1: Daily Dietary Consumption by Sex Under Non-Choice Diet Treatment

We measured the daily intake of each prey insect (ant or cricket) during a non-choice feeding test to estimate the average nutritional intake per day. Ten adult tiger beetles (five females, five males) were used per non-choice diet treatment, with a total number of 10 prey insects provided per tiger beetle daily.

Tiger beetles hunt live prey but also scavenge in nature [28]. Thus, to avoid confounding effects of hunting effort on energy expenditure, prey insects were frozen at −20 °C before being offered to the tiger beetles. After 24 h, the number of prey insects consumed was recorded (since the tiger beetle does not completely consume prey insects, if there were traces of the tiger beetle eating the prey insects, it was considered to have been eaten), and any remaining carcasses were removed before fresh prey insects were provided. Daily dietary intake was measured for up to 12 days, and prey insects were provided until the tiger beetle’s death under non-choice diet treatment, allowing us to estimate changes in fecundity and longevity for each sex (Raw Data S1). All tiger beetles were reared individually until the end of the experiment, except for the time required for mating.

#### 2.3.2. Experiment 2: Daily Dietary Consumption by Sex Under Choice Diet Treatment

We sought to identify the prey-insect preferences of tiger beetles and to evaluate whether they optimize their diet when offered a choice between ants and crickets, independently of non-choice diet treatment.

Ten adult tiger beetles (five females, five males) were used in the choice diet treatment, with each tiger beetle provided with ten prey insects daily (five ants and five crickets). After 24 h, the numbers of each prey insect consumed were recorded, and remaining carcasses were removed and replaced with new prey insects. Daily consumption was measured for up to 20 days, and preference for each prey type was analyzed by sex (Raw Data S2). All tiger beetles were reared individually until the end of the experiment, except for the time required for mating.

### 2.4. Measurement of Fecundity and Longevity

Fecundity was measured as the total number of eggs laid by each female throughout her lifespan in the non-choice diet treatment. Females were randomly paired with males from the same diet treatment once a week to allow mating. Mating was performed by placing one male and one female in the single-rearing jar, and then separating them again after mating occurred within 8 h. We counted eggs weekly by transferring the sea sand from each rearing jar onto a stainless-steel tray, allowing for manual inspection and the counting of eggs [26]. Following each egg count and/or mating, each tiger beetle was transferred to a new rearing jar. This procedure was repeated weekly until the end of the experiment. To ensure accurate estimation of dietary intake, data from the days on which mating occurred were excluded from daily intake record, as these procedures did not span a full 24 h cycle.

Longevity was similarly measured under the non-choice diet treatment. Lifespan was defined as the period from collection to death, with the dates of death recorded daily. Variations in lifespan between sexes were compared under different diet treatments.

### 2.5. Data Analyses

Longevity (lifespan) differences between sexes and fecundity variations (total egg production) in female tiger beetles by diet treatments were analyzed using one-way analysis of variance (ANOVA), because all values satisfied the assumption of normal distribution (Shapiro–Wilk tests, *p* > 0.01).

Prey insect preference by each sex under a choice diet treatment was quantified using a preference index, which was calculated as (*N*_(cricket)_ − *N*_(ant)_)/(*N*_(cricket)_ + *N*_(ant)_) × 100%. This index ranges from 100 (absolute preference for the cricket) to –100 (absolute preference for the ant), with 0 indicating no preference. Each prey insect’s preference index was compared to 0 using the Wilcoxon rank-sum test. Five individuals of each sex were observed, the number consumed of each prey insect was measured every 24 h for up to 20 days, and a total number of 100 counts for each prey insect was calculated.

Dietary consumption by sex under non-choice diet treatment and prey-insect preference by sex under choice diet treatment were analyzed using either linear mixed models (LMMs) or generalized linear mixed models (GLMMs), depending on the distribution of the response variables. For the choice diet treatment, we fitted an LMM with the *index for prey-insect preference* as the response variable as it followed a normal distribution. For the non-choice diet treatment, we used GLMMs with the *number of prey items* (daily dietary consumption of ants or crickets) as the response variable, assuming either a Poisson or a negative binomial distribution. In all models, *identity* and *investigation days* were treated as random effects, whereas *diet* or *sex* was included as a fixed effect.

All statistical analyses were conducted in R version 4.3.2. [29]. Data normality was conducted using *Shapiro.test* function. For one-way ANOVA and Wilcoxon rank-sum tests, we used *aov* and *wilcox.test* functions, respectively. For LMMs, we used *lmer* function from the *lme4* and *lmerTest* packages, while *glmmTMB* function from *glmmTMB* package was used for GLMMs. Overdispersion was assessed using the *simulateResiduals* function from the *DHARMa* packages.

## 3. Results

### 3.1. Daily Dietary Consumption Under Non-Choice Diet Treatment

**Figure 2:**
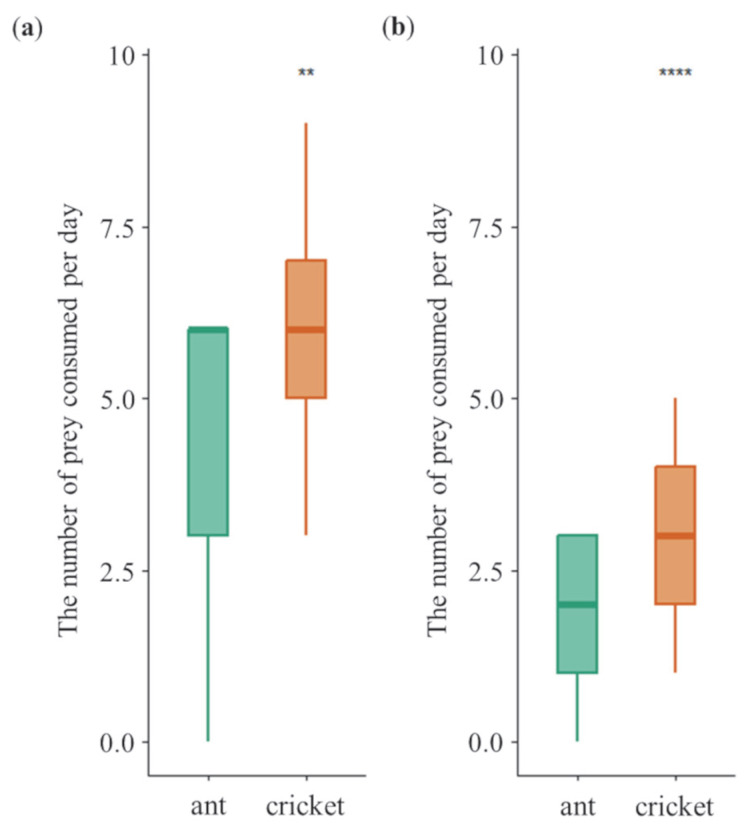
Daily diet consumption of female (**a**) and male (**b**) tiger beetles in the non-choice experiment. Asterisks indicate significant differences between diet treatments (**, *p* < 0.01; ****, *p* < 0.0001). Error bars are standard errors of the mean.

Across sexes, tiger beetles consumed more high-P:C diet (cricket) than low-P:C diet (ant) under non-choice diet treatment (GLMMs, *z* = 5.762, *p* = 8.33 × 10^−9^; Figure 2). Specifically, females consumed more crickets (5.8 ± 1.3 per day) than ants (4.5 ± 2.0 per day; GLMMs, *z* = 3.209, *p* = 1.33× 10^−3^) (Figure 2a). Similarly, males also consumed more crickets (3.2 ± 1.2 per day) than ants (1.7 ± 1.1 per day; GLMMs, *z* = 6.057, *p* = 1.39 × 10^−9^) (Figure 2b). Furthermore, females exhibited significantly higher overall dietary consumption than males under non-choice treatment, supporting *prediction I*.

### 3.2. Longevity of Female and Male

**Figure 3:**
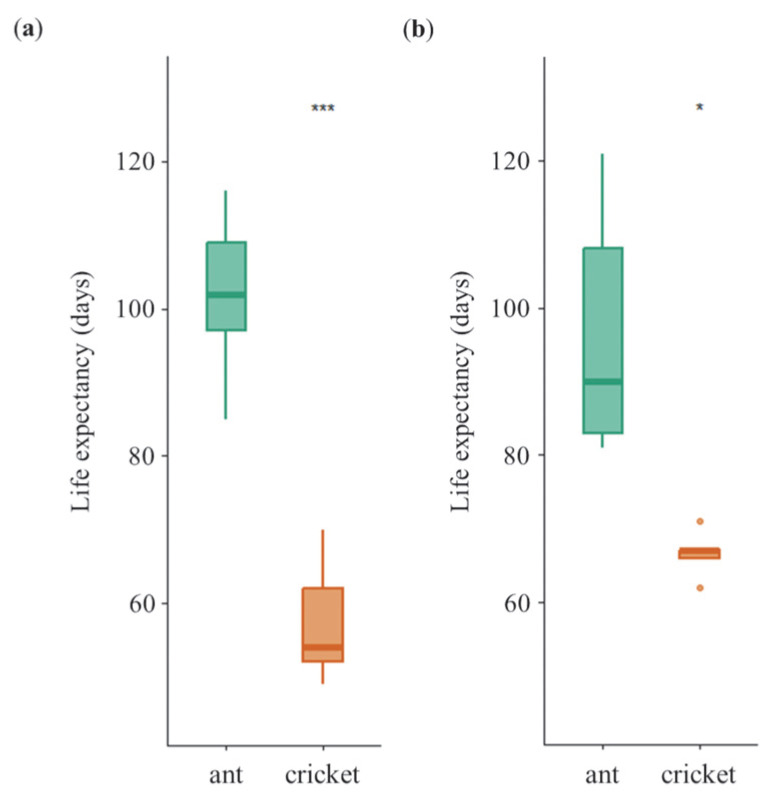
Longevity of female (**a**) and male (**b**) tiger beetles in the non-choice experiment. Asterisks indicate significant differences between diet treatments (*, *p* < 0.01; ***, *p* < 0.001). Error bars are standard errors of the mean.

No significant sex-specific difference in longevity was detected within either diet treatment (one-way ANOVA, ant: *F*_1,18_ = 0.308, *p* = 0.594; cricket: *F*_1,18_ = 5.093, *p* = 0.054) (Figure 3). However, prey-insect type had a significant effect on lifespan. Females fed ants (101.8 ± 11.8 days) lived significantly longer than females fed crickets (57.4 ± 8.5 days; one-way ANOVA, *F*_1,8_ = 46.38, *p* = 1.36 × 10^−4^; Figure 3a). Males showed the same pattern: individuals fed ants (96.6 ± 17.3 days) lived longer than those fed crickets (66.6 ± 3.2 days; one-way ANOVA, *F*_1,8_ = 14.54, *p* = 5.14 × 10^−3^; Figure 3b). These results support *prediction III* regarding dietary effects on longevity.

### 3.3. Fecundity of Female Tiger Beetle

**Figure 4:**
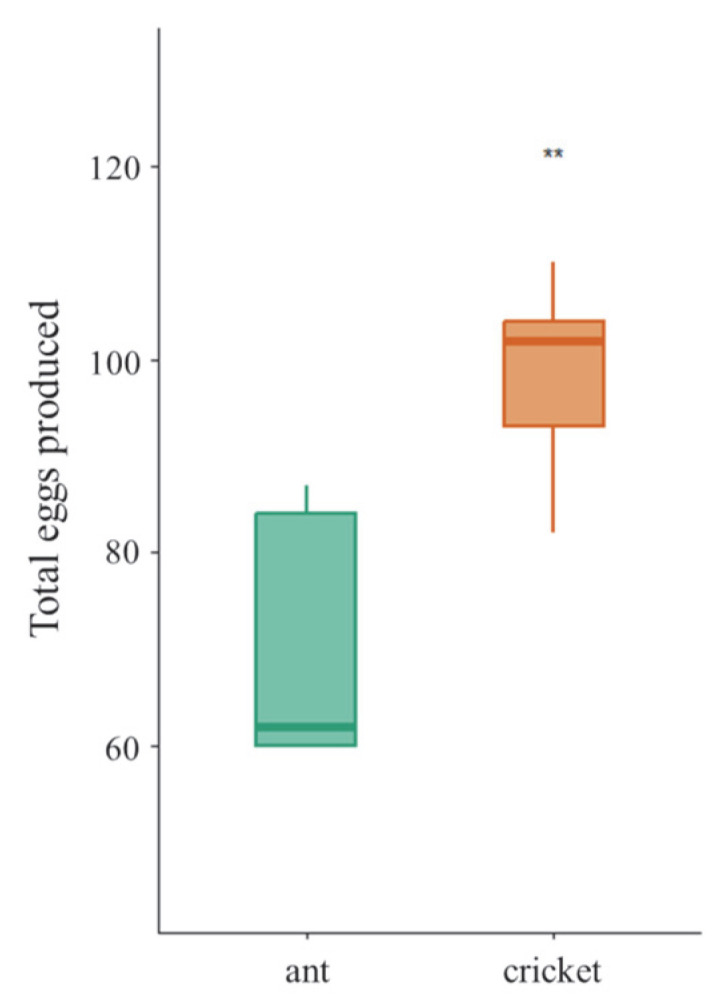
Total eggs produced by females from each diet treatment. Asterisk indicates significant differences between diet treatments (**, *p* < 0.01). Error bars are standard errors of the mean.

Prey insect significantly affected female tiger beetle fecundity. Females fed on crickets produced more eggs (98.2 ± 10.9) than those fed on ants (70.6 ± 13.7; one-way ANOVA, *F*_1,8_ = 12.45, *p* = 7.75 × 10^−3^) (Figure 4). The variations in fecundity were observed depending on the prey insect, and this result explained the contents of *prediction III* regarding dietary effects on fecundity.

### 3.4. Dietary Preference Under Choice Diet Treatment

**Figure 5:**
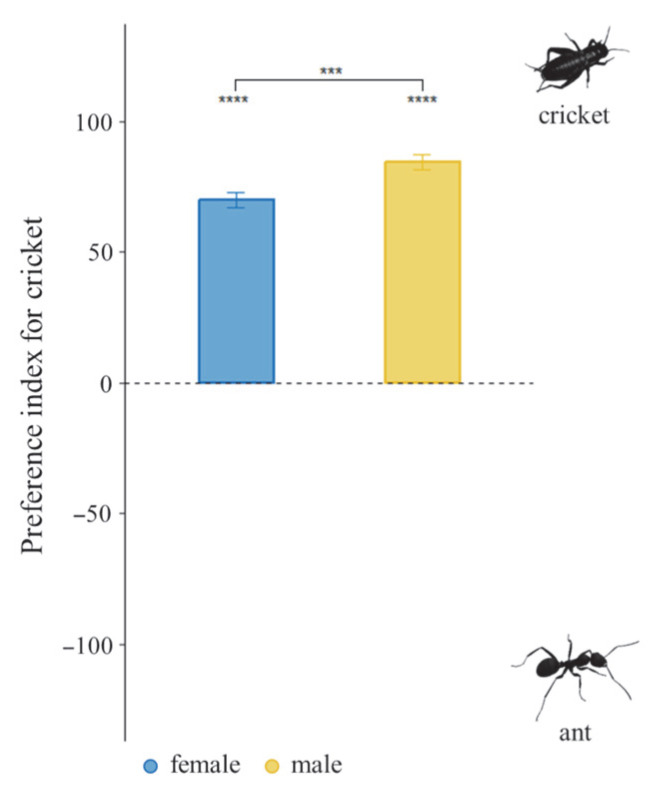
Prey-preference index in the choice experiment (positive = cricket; negative = ant). Asterisks indicate significant differences between diet treatments (***, *p* < 0.001; ****, *p* < 0.0001). Error bars are standard errors of the mean.

**Figure 6:**
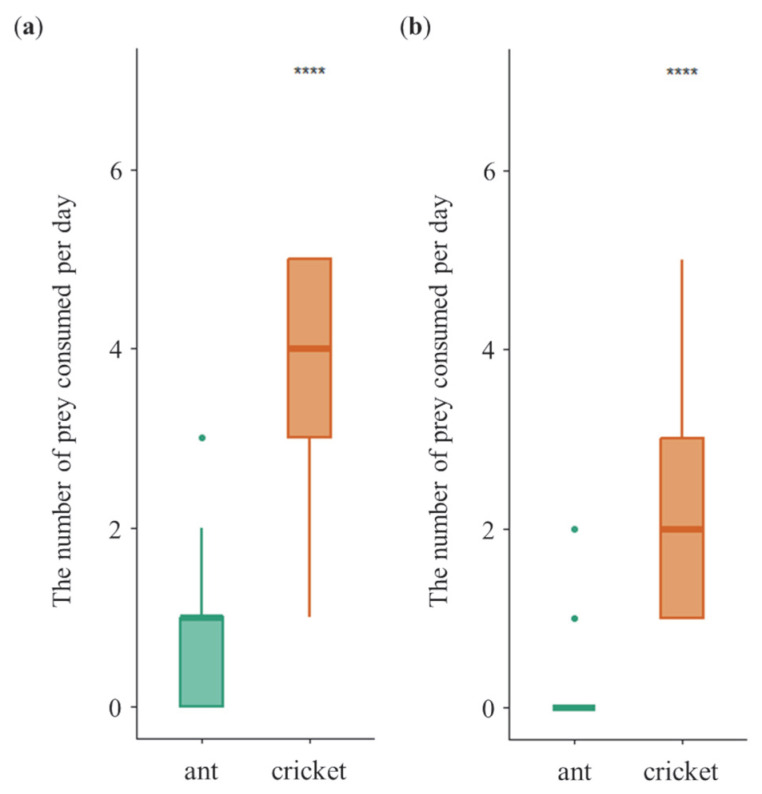
Daily diet consumption of female (**a**) and male (**b**) tiger beetles in the choice experiment. Asterisk indicates significant differences between diet treatments (****, *p* < 0.0001). Error bars are standard errors of the mean.

When offered ants and crickets simultaneously, both sexes showed a significant preference for crickets over ants (LMMs, *t* = 3.089, *p* = 0.0149) (Figure 5). Females consumed more crickets (4.1 ± 1.1 per day) than ants (0.8 ± 0.9 per day; GLMMs, *z* = 13.167, *p* = 2.0 × 10^−16^) (Figure 6a). Similarly, males also consumed more crickets (2.3 ± 1.0 per day) than ants (0.3 ± 0.5 per day; GLMMs, *z* = 10.435, *p* = 2.0 × 10^−16^) (Figure 6b), consistent with patterns observed in the non-choice diet treatment. These results show that tiger beetles have preferences for specific prey insects regardless of sex, supporting *prediction IV*.

## 4. Discussion

Our findings provide empirical evidence that the fecundity–longevity trade-off observed in *C. anchoralis* is influenced by prey insects, even under habitat environmental conditions. Consistent with *prediction I*, female tiger beetles consumed significantly more prey insects than males regardless of prey-insect type. This pattern is also reported in other insect taxa [30,31,32] and may reflect the generally larger body size of females [33], the greater energetic demands of oogenesis [34], or both.

Contrary to *prediction II*, to compensate for nutritional deficiencies, tiger beetles did not consume more ants (low-P:C diet) than crickets (high-P:C diet) under the condition of non-choice diet treatment. Regardless of the type of prey insect, despite nutritional and body mass differences between ants and crickets, daily prey insect consumption of tiger beetles remained relatively consistent: female tiger beetles consumed approximately 5–6 prey insects per day, while male tiger beetles consumed approximately 2–3 prey insects per day. These results suggest that tiger beetles do not compensate for nutritional deficiencies in their diet by consuming larger amounts.

In support of *prediction III*, prey insect significantly affected both longevity and fecundity. Tiger beetles fed on ants (low-P:C diet) exhibited lifespans approximately 40% longer than those fed on crickets (high-P:C diet), while tiger beetles consuming crickets laid 30% more eggs over their lifetime. These patterns indicate a clear trade-off between lifespan and reproductive output modulated by prey insects. Importantly, these results were observed under environmental conditions that were similar to habitat circadian temperature and light, challenging claims that lifespan extension under dietary restriction is an artifact of constant laboratory environments [21,22]. The results of previous studies appear to reflect an artifact of fixed-temperature conditions, overlooking daily and spatial thermal variation in natural habitats and animals’ capacity for behavioral avoidance of stressful conditions [35]. From the perspective of avoidance behavior in response to temperature changes, the tiger beetle also shows thermoregulation behaviors (e.g., stilting, flight, burrowing, drinking) [36]. Nevertheless, since adult insects have very limited mating periods and short lifespans [37], the adaptive resource re-allocation hypothesis according to diet does not seem to be convincing.

An alternative explanation for the reduced lifespan observed in the tiger beetles fed crickets is the physiological cost of detoxification. Certain amino acids found in protein-rich diets, such as methionine, have been shown to reduce lifespan in other insects through toxic effects [6,13]. Recent studies also indicate that the development of the reproductive organs in *Drosophila* is not delayed under low-P:C diets, further weakening the notion that dietary restriction triggers somatic prioritization [38,39]. It is plausible, therefore, that the high-P:C diet shortens lifespan due to the cost of detoxifying amino acid excesses, while extended lifespan on the low-P:C diet may result from reduced physiological stress. Further biochemical and physiological research is needed to elucidate the exact mechanisms underlying this trade-off.

Under choice diet treatment, supporting *prediction IV*, tiger beetles exhibited a strong and consistent preference for crickets, despite its associated survival cost. This behavior contradicts the expectation—predicted by the geometric framework for nutrition—that animals will self-select nutritionally balanced diets to optimize fitness [40]. Instead, tiger beetles appear to prefer prey insects that promote rapid reproductive output, potentially reflecting a life-history strategy shaped by high extrinsic mortality in natural habitats (e.g., predation, infection, disturbance). This prey-insect preference supports a faster-life strategy in which individuals maximize early fecundity at the expense of longevity [41], and it appears to prioritize population-level reproductive success over individual longevity, which may have resulted in increases in intrinsic rate of natural increase.

These findings have direct implications for ex situ conservation and captive propagation of endangered tiger beetles. High-P:C diets may be particularly advantageous in breeding programs aiming to maximize reproductive output over short time frames. First, increased fecundity can accelerate mass propagation—essential for population restoration [42]—and can be achieved through appropriate dietary provision [43]. Second, shortened lifespans may lead to more synchronized oviposition, facilitating cohort management. Third, aligning the reproductive physiology of endangered tiger beetle species with that of short-lived, highly fecund model organisms (e.g., mice, fish, fruit flies, and nematodes) could reduce the time, labor, and costs associated with breeding programs [44]. However, this study was limited by the inability to conduct experiments on a large number of tiger beetles due to the species being endangered. To achieve more accurate statistical analysis results, further research using tiger beetles secured in large quantities through ex situ conservation methods would be necessary.

## 5. Conclusions

This study demonstrates that prey insect shapes key life-history traits in *C*. *anchoralis*, with cricket (high-P:C diet) intake enhancing fecundity but reducing lifespan. These trade-offs persist under ecologically realistic conditions and are likely to reflect evolved strategies rather than laboratory artifacts. Despite the survival cost, *C*. *anchoralis* preferentially consume crickets and does not behaviorally compensate for nutritional imbalance, suggesting an evolved prioritization of early reproductive output. This faster-life strategy may enhance population-level fitness under high extrinsic mortality. Together, these findings provide a valuable framework for developing targeted dietary protocols in conservation breeding programs to support the recovery of endangered tiger beetles.

## Figures and Tables

**Table 1:** Nutritional contents of the two prey insects used in the experiments.

Prey Insects	Body Size (mm) *^b^*	Body Weight (mg) *^c^*	Nutritional Contents *^a^*							P:C Ratio *^d^*
			Energy Content (kcal/100 g)	Sodium (mg/100 g)	Carbohydrate (g/100 g)	Sugars (g/100 g)	Crude Fat (g/100 g)	Saturated Fat (g/100 g)	Crude Protein (g/100 g)	
Japanese wood ant	5.5 ± 0.1	2.9 ± 0.4	136.66	98.40	6.22	1.12	3.34	0.39	20.43	3.28:1
Two-spotted cricket	6.3 ± 0.1	5.0 ± 0.4	123.18	102.88	3.03	0.13	4.18	0.90	18.36	6.06:1

*^a^* For analysis of nutrients, two prey species were analyzed externally by the Korea Standard Test and Analysis Research Institute, Ansan-si, South Korea. *^b,c^* Body size and weight were calculated as the mean and standard error of wet weight of 10 ant workers and 10 cricket 2nd nymphs, respectively. *^d^* The P:C ratio was calculated as crude protein divided by carbohydrate.

## Data Availability

The original contributions presented in this study are included in the article/Supplementary Material. Further inquiries can be directed to the corresponding author.

## Supplementary Materials

The following supporting information can be downloaded at https://www.mdpi.com/article/10.3390/insects16101066/s1. Raw Data S1: the information of the individual *C*. *anchoralis* used in non-choice diet treatment, along with their daily dietary intake, egg-production, and lifespan records. Raw Data S2: the information of the individual *C*. *anchoralis* used in choice diet treatment, along with the types and amounts of prey insect consumed daily, and PI (preference index) value.

## References

[1] Weindruch R., Walford R. (1982). Dietary restriction in mice beginning at 1 year of age: Effect on life-span and spontaneous cancer incidence. Science.

[2] Fontana L., Partridge L., Longo V.D. (2010). Extending healthy life span-from yeast to humans. Science.

[3] Le Couteur D.G., Solon-Biet S., Cogger V.C., Mitchell S.J., Senior A., de Cabo R., Raubenheimer D., Simpson S.J. (2016). The impact of low-protein high-carbohydrate diets on aging and lifespan. Cell. Mol. Life Sci..

[4] Lee K.P., Simpson S.J., Clissold F.J., Brooks R., Ballard J.W.O., Taylor P.W., Soran N., Raubenheimer D. (2008). Lifespan and reproduction in *Drosophila*: New insights from nutritional geometry. Proc. Natl. Acad. Sci. USA.

[5] Roeder K.A., Behmer S.T. (2014). Lifetime consequences of food protein-carbohydrate content for an insect herbivore. Funct. Ecol..

[6] Arganda S., Bouchebti S., Bazazi S., Le-Hesran S., Puga C., Latil G., Simpson S.J., Dussutour A. (2017). Parsing the life-shortening effects of dietary protein: Effects of individual amino acids. Proc. R. Soc. B.

[7] Sultanova Z., Ivimey-Cook E.R., Chapman T., Maklakov A.A. (2021). Fitness benefits of dietary restriction. Proc. R. Soc. B.

[8] Solon-Biet S.M., Walters K.A., Simanainen U.K., McMahon A.C., Ruohonen K., Ballard J.W.O., Raubenheimer D., Handelsman D.J., Le Couteur D.G., Simpson S.J. (2015). Macronutrient balance, reproductive function, and lifespan in aging mice. Proc. Natl. Acad. Sci. USA.

[9] May C.M., Doroszuk A., Zwaan B.J. (2015). The effect of developmental nutrition on life span and fecundity depends on the adult reproductive environment in *Drosophila melanogaster*. Ecol. Evol..

[10] Zajitschek F., Zajitschek S.R.K., Canton C., Georgolopoulos G., Friberg U., Maklakov A.A. (2016). Evolution under dietary restriction increases male reproductive performance without survival cost. Proc. R. Soc. B.

[11] Rho M.S., Lee K.P. (2016). Balanced intake of protein and carbohydrate maximizes lifetime reproductive success in the mealworm beetle, *Tenebrio molitor* (Coleoptera: Tenebrionidae). J. Insect Physiol..

[12] Collins D.H., Prince D.C., Donelan J.L., Chapman T., Bourke A.F.G. (2023). Developmental diet alters the fecundity–longevity relationship and age-related gene expression in *Drosophila melanogaster*. J. Gerontol. A Biol. Sci. Med. Sci..

[13] Rau V., Flatt T., Korb J. (2023). The remoulding of dietary effects on the fecundity/longevity trade-off in a social insect. BMC Genom..

[14] McGowan P.J.K., Traylor-Holzer K., Leus K. (2017). IUCN guidelines for determining how ex situ management should be used in species conservation. Conserv. Lett..

[15] Atkinson J., Bonser S.P. (2020). “Active” and “passive” ecological restoration strategies in meta-analysis. Restor. Ecol..

[16] Fardisi M., Mason L.J., Ileleji K.E. (2013). Development and fecundity rate of *Tribolium castaneum* (Herbst) on distillers dried grains with solubles. J. Stored Prod. Res..

[17] Pan Q., Ang Y., Shikano I. (2024). Effects of adult diet on the longevity, fecundity and ovarian development of the rice leaffolder, *Cnaphalocrocis medinalis*. Physiol. Entomol..

[18] Millar J.G., Paine T.D., Joyce A.L., Hanks L.M. (2003). The effects of *Eucalyptus* pollen on longevity and fecundity of *Eucalyptus* longhorned borers (Coleoptera: Cerambycidae). J. Econ. Entomol..

[19] Chen E.H., Wei D., Wei D.D., Yuan G.R., Wang J.J. (2013). The effect of dietary restriction on longevity, fecundity, and antioxidant responses in the oriental fruit fly, *Bactrocera dorsalis* (Hendel) (Diptera: Tephritidae). J. Insect Physiol..

[20] Su S., Zhang X., Jian C., Huang B., Peng X., Vreysen M.J.B., Chen M. (2022). Effects of adult feeding treatments on longevity, fecundity, flight ability, and energy metabolism enzymes of *Grapholita molesta* Moths. Insects.

[21] Adler M.I., Bonduriansky R. (2014). Why do the well-fed appear to die young? A new evolutionary hypothesis for the effect of dietary restriction on lifespan. Bioessays.

[22] Zajitschek F., Zajitschek S.R.K., Vasconcelos A.C.O., Bonduriansky R. (2023). Dietary restriction fails to extend life in stressful environments. Funct. Ecol..

[23] Chevrolat L.A.A. (1845). Description of ten beetles from China, from the surroundings of Macao, and from an acquisition made at M. PARSUDAKI, naturalist dealer in Paris. Zool. Rev. Cuvierian Soc..

[24] Suh M.H. (2023). Red Data Book of Republic of Korea Volume 8, Insecta II.

[25] Kim C.W. (1978). Distribution Atlas of Insects of Korea, Series 2, Coleoptera.

[26] Cha D., Jung J.K. (2024). Captive propagation and observations of the endangered species *Cicindela* (*Abroscelis*) *anchoralis* (Coleoptera: Carabidae: Cicindelinae) in South Korea. Insect. Conserv. Divers..

[27] Lee K.P. (2015). Dietary protein:carbohydrate balance is a critical modulator of lifespan and reproduction in *Drosophila melanogaster*: A test using a chemically defined diet. J. Insect Physiol..

[28] Schultz T.D. (1981). Tiger beetles scavenging on dead vertebrates. Cicindela.

[29] R Core Team (2023). R: A Language and Environment for Statistical Computing.

[30] Gutiérrez Y., Fresch M., Ott D., Brockmeyer J., Scherber C. (2020). Diet composition and social environment determine food consumption, phenotype and fecundity in an omnivorous insect. R. Soc. Open Sci..

[31] Moeser J., Vidal S. (2005). Nutritional resources used by the invasive maize pest *Diabrotica virgifera virgifera* in its new South-east-European distribution range. Entomol. Exp. Appl..

[32] Mevi-Schütz J., Goverde M., Erhardt A. (2003). Effects of fertilization and elevated CO_2_ on larval food and butterfly nectar amino acid preference in *Coenonympha pamphilus* L. *Behav*. Ecol. Sociobiol..

[33] Yadav P., Patel P., Patel A.K., Chowdhury R., Upadhyay A., Kumar B., Kumar D. (2024). Ageing and mating status affect food utilization efficiencies and assimilation of macronutrients in adults of Parthenium beetle, *Zygogramma bicolorata* Pallister. Physiol. Entomol..

[34] Wheeler D. (1996). The role of nourishment in oogenesis. Annu. Rev. Entomol..

[35] Ma C.S., Ma G., Pincebourde S. (2021). Survive a Warming Climate: Insect Responses to Extreme High Temperatures. Annu. Rev. Entomol..

[36] Dreisig H. (1979). Daily activity, thermoregulation and water loss in the tiger beetle *Cicindela hybrid*. Oecologia.

[37] Mautz B.S., Rode N.O., Bonduriansky R., Rundle H.D. (2019). Comparing ageing and the effects of diet supplementation in wild vs. captive antler flies, *Protopiophila litigata*. J. Anim. Ecol..

[38] Mair W., Sgrò C.M., Johnson A.P., Chapman T., Partridge L. (2004). Lifespan extension by dietary restriction in female *Drosophila melanogaster* is not caused by a reduction in vitellogenesis or ovarian activity. Exp. Gerontol..

[39] Drewry M.D., Williams J.M., Hatle J.D. (2011). Life-extending dietary restriction and ovariectomy result in similar feeding rates but different physiologic responses in grasshoppers. Exp. Gerontol..

[40] Nakagawa S., Lagisz M., Hector K.L., Spencer H.G. (2012). Comparative and meta-analytic insights into life extension via dietary restriction. Aging Cell.

[41] Ellis B.J., Figueredo A.J., Brumbach B.H., Schlomer G.L. (2009). Fundamental Dimensions of Environmental Risk. Hum. Nat..

[42] Knisley C.B., Hill J.M., Scherer A.M. (2005). Translocation of threatened tiger beetle *Cicindela dorsalis dorsalis* (Coleoptera: Cicindelidae) to Sandy Hook, New Jersey. Ann. Entomol. Soc. Am..

[43] Bertinetti C., Samayoa A.C., Hwang S.Y. (2019). Effects of feeding adults of *Hermetia illucens* (Diptera: Stratiomyidae) on longevity, oviposition, and egg hatchability: Insights into optimizing egg production. J. Insect. Sci..

[44] Holtze S., Gorshkova E., Braude S., Cellerino A., Dammann P., Hildebrandt T.B., Hoeflich A., Hoffmann S., Koch P., Terzibasi Tozzini E. (2021). Alternative Animal Models of Aging Research. Front. Mol. Biosci..

